# A Comprehensive Review on the Surgical Aspect of Lung Transplant Models in Mice and Rats

**DOI:** 10.3390/cells11030480

**Published:** 2022-01-30

**Authors:** Xin Jin, Janne Kaes, Jan Van Slambrouck, Ilhan Inci, Stephan Arni, Vincent Geudens, Tobias Heigl, Yanina Jansen, Marianne S. Carlon, Robin Vos, Dirk Van Raemdonck, Yi Zhang, Bart M. Vanaudenaerde, Laurens J. Ceulemans

**Affiliations:** 1 Laboratory of Respiratory Diseases and Thoracic Surgery (BREATHE), Department CHROMETA, KU Leuven, 3000 Leuven, Belgium; xin.jin@student.kuleuven.be (X.J.); janne.kaes@kuleuven.be (J.K.); jan.vanslambrouck@kuleuven.be (J.V.S.); vincent.geudens@kuleuven.be (V.G.); tobias.heigl@kuleuven.be (T.H.); yaninajansen@gmail.com (Y.J.); marianne.carlon@kuleuven.be (M.S.C.); robin.vos@uzleuven.be (R.V.); dirk.vanraemdonck@uzleuven.be (D.V.R.); bart.vanaudenaerde@kuleuven.be (B.M.V.); 2 Department of Thoracic Surgery, University Hospitals Leuven, 3000 Leuven, Belgium; 3 Department of Thoracic Surgery, University Hospital Zürich, 8091 Zürich, Switzerland; Ilhan.inci@usz.ch (I.I.); stephan.arni@usz.ch (S.A.); 4 Department of Pharmaceutical and Pharmacological Sciences, Molecular Virology and Gene Therapy, KU Leuven, 3000 Leuven, Belgium; 5 Department of Respiratory Diseases, University Hospitals Leuven, 3000 Leuven, Belgium; 6 Department of Thoracic Surgery, Xuanwu Hospital, Capital Medical University, Beijing 100053, China

**Keywords:** lung transplantation, small animal model, mouse, rat, rodent, microsurgery

## Abstract

Lung transplantation improves the outcome and quality of life of patients with end-stage pulmonary disease. However, the procedure is still hampered by the lack of suitable donors, the complexity of the surgery, and the risk of developing chronic lung allograft dysfunction. Over the past decades, translational experiments in animal models have led to a better understanding of physiology and immunopathology following the lung transplant procedure. Small animal models (e.g., rats and mice) are mostly used in experiments regarding immunology and pathobiology and are preferred over large animal models due to the ethical aspects, the cost–benefit balance, and the high throughput possibility. In this comprehensive review, we summarize the reported surgical techniques for lung transplantation in rodent models and the management of perioperative complications. Furthermore, we propose a guide to help identify the appropriate species for a given experiment and discuss recent experimental findings in small animal lung transplant models.

## 1. Introduction

Lung transplantation (LTx) is the ultimate curative treatment for end-stage pulmonary disease. Around 70,000 lung transplant procedures have been reported worldwide to the International Society for Heart and Lung Transplantation (ISHLT). Importantly, long-term survival after LTx still lags behind compared to other solid organ transplantations, with a 5-year survival rate of only 59% [1,2].

The low survival rate is the result of the surgical complexity, primary graft dysfunction (PGD), chronic lung allograft dysfunction (CLAD), and the side effects of the life-long immunosuppressive therapy such as infections and cancer [2]. Therefore, preclinical animal models are essential to elucidate immunological and pathophysiological processes and to test new therapeutic strategies. Over the past decades, progress has been made based on findings obtained from various animal models (e.g., rodent, dog, non-human primate, and porcine) [3,4,5].

## 2. The Advantage of Rodent Models

The ideal animal model should provide strong physiological and anatomical similarity to the human disease process, and the model should be balanced between resource and cost. Unfortunately, the ideal model does not exist. Depending on the research objectives, a choice should be made after surveying the advantages and disadvantages of a particular model. Elements that should be taken into consideration are outlined in Table 1.

A mouse or rat model is cost-efficient, as rodents are easy to house and have a short gestation period. Therefore, large sample sizes can be reached in a short timeframe and at a low cost. These advantages increase the replicative value in research leading to more robust and credible results. However, various rodent models are used with different advantages. Compared to mice, rats are larger, making experimental surgery less technically demanding. On the other hand, immunological read-outs in mice are more documented, with numerous assays (transcriptomics, ELISA, flow cytometry) to quantify the immunological response after LTx [6,7]. Additionally, mice can be easily genetically modified to create transgenic strains, assessing the effect of specific gene knock-outs related to pathophysiological processes that underlie (un)successful LTx [8]. To study chronic diseases, like chronic rejection after LTx, the short lifespan and high metabolic rate of rodents are advantageous compared to other models. 

Considering the physiological and anatomical similarities, pigs are more closely related to humans than rodents. For this purpose, research in pigs has a stronger translational value. However, experiments with pigs are demanding in required personnel (e.g., surgeons, veterinarians, animal caretakers) and facilities. It is challenging to study immunological processes in outbred transplant pigs due to the higher sensibility to infection and higher variability. The longer lifespan and slower metabolic rate result in difficulties in observing chronic processes after LTx. Accordingly, rodents should be the first step to study a hypothesis, including several experimental groups, which could be later confirmed with a smaller study design in pigs.

## 3. Choosing the Best Species for a Given Experiment 

Obviously, the size is one of the most significant differences between rats and mice (Table 1). Due to this difference, the operation field in rats is larger, rendering the surgery less complex than in mice. Therefore, more complicated techniques such as ex vivo lung perfusion (EVLP) and retransplant experiments can be applied in rat LTx, while these are difficult to perform in mice. Additionally, serial blood sampling with larger volumes will be more feasible in rats, as they have a higher circulatory blood volume of approximately 14–20 mL per 250–300 g of body weight versus mice with 1–1.8 mL total blood volume per 20–30 g body weight [9]. 

Although the purchase costs for mice and rats are approximately similar, housing costs are slightly higher for rats as multiple mice (up to five) can be housed together. The most significant advantage in using mice is the availability of much more transgenic models compared to rats [10]. Indeed, there are multiple various knock-out, knock-in, and transgenic strains widely available from different suppliers in mice. For example, the wild-type C57BL/6J background syngeneic mice LTx model can be applied to study the role of *Sprouty-related EVH1 domain-containing protein 2* (*SPRED2*) (WT and *SPRED2*^−/−^ to WT) in protecting the recipient from ischemia–reperfusion injury (IRI) in the transplanted lung [11]. A BALB/c to C57BL/6 model, which resembles the human major HLA mismatch setting, can be used to investigate acute or chronic rejection after LTx. As these mice are inbred, it is easier to extract uniform immunological responses per treatment group. However, there are several different combinations used for donor and recipient strain. Consequently, it is important to be cautious in comparing results obtained from different strain combinations. 

Thus, when choosing the right species, the hypothesis and aim of a specific experiment should be well thought out, and the difference between models should be considered. In addition, different models are available in the same species. For example, right LTx in mice could be a more relevant model for observing pulmonary function changes, as it can still survive after a left pneumonectomy [12]. However, in the case of left LTx, the right lung may enlarge to compensate for the loss of function of the left transplanted and rejected lung. Therefore, lung function measurement is not feasible in this case [13]. One should also consider the disparities between rodents and humans to extrapolate results from bench to bedside. The main difference between rodent and human lungs is that rodents lack respiratory bronchioles, as the terminal bronchioles empty immediately into the alveolar ducts [14]. This implies several implications. For example, obliterative bronchiolitis (OB), which is the histological hallmark of bronchiolitis obliterans syndrome (BOS) in humans, characterized by obliteration of the respiratory bronchioles, cannot be observed in rodents, making them a less suitable model for this research [15,16].

## 4. Surgical LTx Procedure 

### 4.1. Rat Donor Organ Procurement

The orthotopic (transplantation of an organ into its normal position) single-lung transplantation is more frequently conducted using the left lung because it is one complete entity, whereas the right lung has four lobes. The donor and recipient should be matched in body shape, but choosing a smaller donor may provide convenience in anastomosis to a certain extent. The donor rat can be anesthetized with an intraperitoneal injection of ketamine (100 mg/mL) and xylazine (20 mg/mL) or with a mixture of oxygen and isoflurane depending on facilities and local regulations. The donor is positioned in supine position. A tracheotomy is performed, and an endotracheal tube is inserted and connected to the ventilator under a volume-controlled (VC) setting. The ventilator’s tidal volume (TV) is set to 5–10 mL/kg, respiratory rate (RR) at 60–100/min, positive end-expiratory pressure (PEEP) at 2–4 cm H_2_O, and fraction of inspiration O₂ (FiO_2_) at 0.5–1.0 [17,18,19].

**Figure 1:**
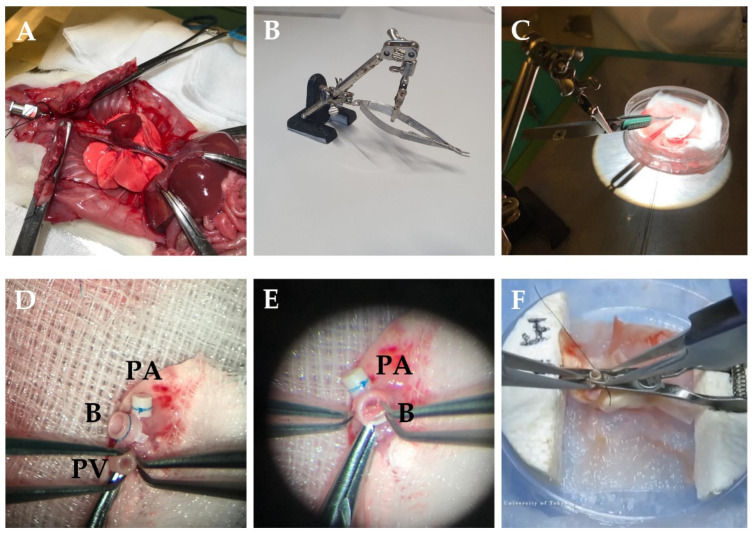
Donor procedure in rats LTx. (**A**) The chest wall is flipped outward with forceps and fixed on both sides. (**B**) Stabilizer with needle holder. (**C**) Benchwork for preparing donor lung. (**D**) The vessel is passed through the cuff, and tissue is folded over the cuff body and secured with a 7-0 nylon suture. (**E**) The bronchus is cuffed the same way as the vessels. (**F**) The bulldog clamp is set into foam blocks as a stabilizer for cuffing vessels and bronchus [25]. PV—pulmonary vein; B—bronchus; PA—pulmonary artery.

The laparotomy is performed, the xyphoid is dissected and retracted cephalad to expose the diaphragm. The chest wall is flipped outward with forceps and fixed on both sides to expose the whole thorax (Figure 1A). The inferior vena cava is injected with 500–1000 IU/kg heparin. After heparinization, the inferior vena cava and left atrium are incised for exsanguination. Perfusion is performed with 10–20 mL 4 °C low-potassium dextran-glucose solution into the pulmonary artery (PA) at a constant pressure of 20–30 cm H_2_O (similar to the physiologic PA systolic pressure in rats) [20,21,22]. Perfusion needs to take place while the lungs are ventilated as this ensures flushing of the small capillaries. The chest cavity should be filled with blood during perfusion to avoid air emboli going through the pulmonary valve. After the perfusion, the trachea is clamped while the lung remains inflated with 50% O_2_. A whole heart–lung bloc extraction is recommended when harvesting the donor. At this moment, the heart–lung bloc can be dissociated from surrounding tissue and esophagus. The lungs are placed on a petri dish with gauze soaked in cold low-potassium dextran-glucose solution. 

### 4.2. Cuff Technique for Anastomosis

Since first introduced in 1989, the cuff technique has been modified several times and applied worldwide in small animal models because it can dramatically reduce the technical complexity of performing the anastomoses and subsequent postoperative complications after LTx [23]. The cuffs are made of intravenous catheters. For a donor rat weighing approximately 300 g, a 16–14-gauge cuff is used for the bronchus (B) and 18–16-gauge cuffs for the PA and pulmonary vein (PV). The surface of the cuff can be roughened with sandpaper to avoid the tissue from sliding back [17,24].

To maintain the operation in a hypothermic humid environment and to reduce the organ’s warm ischemia time, the petri dish is placed on ice, and the organ is covered with wet gauze. The left hilum is dissected carefully to dissociate the PA, PV, and bronchus. Then, the heart and right lung are removed from the donor’s left lung. A needle holder is used to secure the cuff (Figure 1B,C). The vessel is passed through the cuff, and tissue is folded over the cuff body and secured with a 7-0 nylon suture. The bronchial cuff is inserted the same way as the vessels (Figure 1D,E). Moreover, there is a technique by using a petri dish with carved foam blocks and a bulldog clamp. The bulldog clamp is set into foam blocks as a stabilizer for cuffing vessels and bronchus, which brings convenience during the cuffing procedure (Figure 1F) [25]. The average duration of these steps is approximately 20 min. The donor lung should be preserved in the low-potassium dextran glucose solution at 4 °C while keeping the bronchus clamped until the transplantation.

### 4.3. Transplantation

It is recommended to use inhalation anesthesia for the recipient as the depth of anesthesia is easy to control by adjusting the anesthetic dose. Isoflurane 1.5–2% or sevoflurane 2.5–3% can be used with 0.01–0.05 mg/kg buprenorphine subcutaneous injection [17,26,27]. Previous studies have also supported the protective effect of pretreatment with inhalation halothane-kind anesthetics, which can attenuate direct severe lung injury [27]. The alternative way is to inject a mixture of 100 mg/kg ketamine and 4–10 mg/kg xylazine intraperitoneal, which requires fewer facilities and costs [28,29,30]. However, it may result in a less stable model because of ongoing low-flow ischemia during the experiment [31].

The depth of anesthesia and analgesia must be checked before incision to reduce the intraoperative usage of isoflurane and its adverse effect on cardiac function. The anesthesia is maintained with isoflurane or sevoflurane or a combination of 15 mg/kg/h ketamine and 1.5 mg/kg/h xylazine to the corresponding anesthesia method. 1.5 mL/h saline is administered subcutaneously or intraperitoneally for volume replacement if the procedure continues for an extended period [30]. 

After the anesthesia induction, the recipient is intubated with an endotracheal tube (ETT) under the guidance of a light source. The size of the used ETT should be matched with the animal’s weight to ensure the best result of ventilation and to avoid either over-pressurizing or air leakage [17,30,32]. The ETT is connected to the ventilator under the VC model with the same settings as the donor procedure. 

The recipient is placed in the right decubitus position on a heating pad. The thoracotomy is performed layer by layer from the area of the cardiac apex impulse, extended dorsally along the ribs. The thorax is entered through the third or fourth intercostal space, the ribs are spread with the retractor, and the left lung is carefully retracted. After dissecting the hilum, the vessels and bronchus are separately clipped near the heart with two clamps or slipknots to expose the anastomosis area. Two aneurysm clips can be used to clamp the PA and PV separately. A T-shape or V-shape incision instead of a simple lateral incision could enlarge the entrance of the cuffed tissues, thereby reducing the tangential friction and the risk of lacerations. All manipulations should be made with fine instruments (e.g., mosquito clamp and eye scissors). To avoid over-inflation of the contralateral lung, one has to adjust TV and RR once the hilum of the left lung is clipped. 

The order of the anastomoses may vary between groups. We prefer to perform the anastomosis in B-A-V order because the connected bronchus could serve as an axis and restrict a torsion of the donor hilum, which could prevent the tearing of fragile vessels. However, the order in V-A-B is also commonly applied. PV anastomosis is the most difficult due to the fragility of the venous wall. Connecting the vessels first can decrease the risk of twisting. A comparison of anastomosis order is summarized in Table 2 [25,33,34,35,36].

The sutures (8-0) are placed around the recipient’s bronchus or vessels, after which the donor structures are passed into the recipient by holding the cuff tail. After securing the anastomosis with thread knots, the clamps/slipknots are released to induce reperfusion (release the PV clip first if clamped separately). The donor lung should always be wrapped with cold soaked gauze during the whole anastomosis. Rat LTx could be mastered after approximately 30–50 transplants [33,37].

After reperfusion, the native lung is removed, and the deflated transplanted donor lung is placed back into the chest cavity with sufficient irrigation. The thorax is closed by placing one interrupted 4-0 Prolene suture around the ribs [32]. The muscles and subcutaneous tissue are sewn interruptedly layer by layer using 4-0 Prolene and Vicryl suture, and the anesthesia is gradually stopped [33]. When the last stitch is placed, one large breath is administrated to recruit the transplanted lung and to eliminate the remaining air and fluid from the chest; hereafter, the chest can be closed completely. The ETT can be removed once the rat breathes spontaneously and is starting to recover. From 0.01 to 0.05 mg/kg buprenorphine should be injected subcutaneously every eight hours until two days after surgery. The subcutaneous application of non-steroid anti-inflammatory drugs such as 5 mg/kg/d ketorolac or 2 mg/kg/d meloxicam relieves postoperative pain and decreases the incidence of anastomotic thrombosis. Antibiotics are not necessary unless the aim is long-term observation [38,39].

### 4.4. LTx in Mice—Similarities and Differences to the Rat Model 

The procedure for mice transplantation is comparable to rats due to the similar anatomy. However, drug dosage and instruments size should be adjusted according to the weight. When extracting the heart-lung bloc, the donor lung is perfused with 2–3 mL 4 °C low-potassium dextran glucose solution at 10cm H_2_O pressure [40]. The PA cuff is made of a 26–24-gauge intravenous catheter, while the PV and B cuff are constructed with a 22–20-gauge catheter [12,41]. Preferably, the tails are removed after cuffing. A 20-gauge ETT is used to intubate mice, and the ventilator settings are TV 0.3–1 mL, RR 120–130/min, and PEEP 0.5 cm H_2_O [41,42,43,44]. 

**Figure 2:**
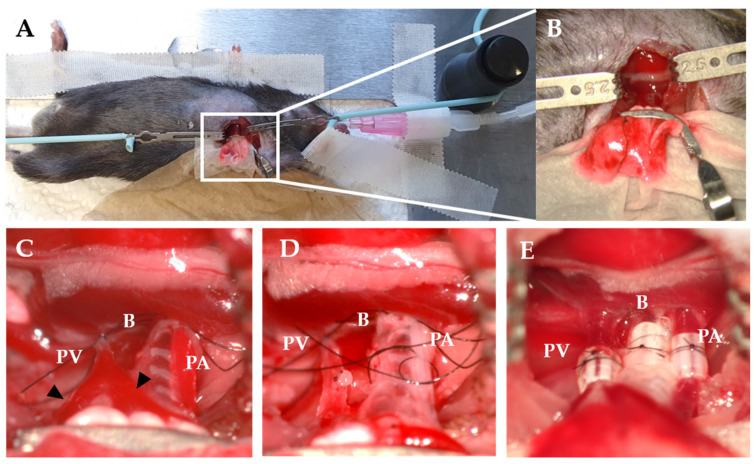
Recipient procedure in mice LTx. (**A**) Right decubitus position of recipient. (**B**) The hilum is exposed using a curved micro serrefine. (**C**) Occlusion of PV and PA using a slipknot. Triangles indicate the branches of the PV. (**D**) B and PA are ligated with a circumferential suture. (**E**) Cuffs after implantation and reperfusion. PV—pulmonary vein; B—bronchus; PA—pulmonary artery.

The thoracotomy is similarly performed in mice as in rats. After the left lung is retracted, a curved micro serrefine is placed on the recipient‘s left lung (Figure 2A,B). The pulmonary artery and vein are closed using 9-0 sutures by making a slipknot (Figure 2C). As the pulmonary vein is most prone to rupture, it might be considered to occlude one of the two branches of the vein to create a larger segment of the recipient’s vein to introduce the donor cuff. To secure the anastomosis, circumferential 10-0 nylon sutures are used for the vessels and 9-0 sutures for bronchus (Figure 2D). After the implantation, the slipknot of the vein is released first, followed by the slipknot of the artery (Figure 2E). 

## 5. Common Complications during and after Orthotopic Left LTx

Complications can arise immediately after implantation. The donor artery could be twisted around its axis before implantation, causing arterial occlusion and blocking reperfusion. It can be resolved by occluding the recipient’s artery again and implanting the donor artery a second time [40]. The donor vein can also be twisted, resulting in venous congestion. Twisting of the donor vein usually happens during the cuffing of the vein and cannot be resolved after implantation. Therefore, it is important to carefully cuff the structures in a straight fashion during the donor procedure. In addition, keeping the donor vein as short as possible can prevent kinking. 

The most life-threatening complication after surgery is a pneumothorax resulting from a broncho- or parenchyma-pleural fistula. Due to bronchial rupture or damage to the lung surface, a severe air leak will be easily recognized upon graft reperfusion and re-aeration. This issue can be solved by utilizing a 10-0 nylon suture to repair a small hole in the bronchus or tissue glue to seal tiny holes on the lung surface [45]. However, the pneumothorax might also occur after extubation, causing respiratory distress and requiring the animal to be sacrificed. Another complication might be hydrothorax due to excessive irrigation in the thoracic cavity. Therefore, one should be cautious with intraoperative saline irrigation and remove most of the fluid before closing the chest. 

Other complications can occur in the first days after LTx. Primary graft dysfunction (PGD) is a phenomenon of acute lung injury after LTx characterized by alveolar edema and hypoxemia. It is observed within the first 72 h after LTx and is mainly caused by IRI [46,47]. The primary method for the prevention of PGD is to shorten the ischemia time. As mentioned above, a homemade operating platform with column and anchors for instrument fixation is strongly recommended to reduce the tension and time of preparing the donor (Figure 1C).

Atelectasis is another common complication occurring in the first days after LTx. The animal may not become symptomatic until it presents with severe respiratory dysfunction. A routine postoperative X-ray or CT should be applied to exclude atelectasis. Common causes of postoperative atelectasis include pleural effusion, pneumothorax, bronchial obstruction, and anastomotic stenosis. For the first two causes, careful hemostasis and donor organ protection can minimize the risk. Adjusting the ventilation in a timely fashion during anesthesia recovery also lowers the risk of pneumothorax. Furthermore, drainage may also be a good preventive measure or treatment method [17,37]. The examination and aspiration of the bronchus before anastomosis minimize the risk of a bronchus obstruction. Choosing a larger B-cuff can enlarge the lumen and reduce airway resistance. The bronchial length of both donor and recipient lungs should be matched. If the anastomosis complex is too long, the bronchus may get occluded or even folded after the donor lung reflates in the closed chest cavity. 

## 6. Recent Findings in LTx on Rodent Models

### 6.1. Ischemia–Reperfusion Injury (IRI) and Ex Vivo Lung Perfusion (EVLP)

IRI induces diffuse endothelial and epithelial damage and pulmonary edema. The injury further causes damage-associated molecular patterns (DAMPs) expression and activates the local innate and adaptive immune system, injuring the graft severely [48] [Reference: Van Slambrouck J. et al. A focus review on primary graft dysfunction after clinical lung transplantation: a multilevel syndrome. Submitted to Cells, Special issue: Advances in Lung Transplantation (2021)]. There are two methods to prevent or relieve the damage: to block the inflammatory pathway expression pre/postoperatively or to shorten the (cold and warm) ischemia time before and during transplantation.

Researchers have found various anti-inflammatory or immune regulatory therapies towards different targets in the small animal LTx model. It has been reported that α-1 antitrypsin (AAT), a plasma serine protease inhibitor, can attenuate acute IRI-induced inflammation and necrosis, indicating AAT’s potential in organ repairment [49,50]. Another example is that post-ischemia neo-epitope C2 is expressed in both mice and human ischemic donor lungs and suggested that targeted treatment could protect donor lungs from complement activation and IRI [51].

The traditional cold organ preservation method shuts down cell energy consumption but also, at the same time, its metabolism and potential for self-repairment. EVLP can maintain the ventilation and circulation of the donor lung ex vivo. Additionally, EVLP also provides opportunities for organ quality assessment and repairing therapies, which is another hot topic in this research field. Wang et al. [52] reported that 3-Aminobenzamide (3-AB) attenuates the IL-6/IL-10 ratio in plasma and bronchoalveolar lavage and maintains the balance between pro- and anti-inflammatory mediators within rat lungs during EVLP. Lonati et al. [53] reported that synthetic α-melanocyte-stimulating hormone analog [Nle4,D-Phe7]-α-MSH (NDP-MSH) during EVLP could exert positive influences in rat lungs exposed to different injuries. On the other hand, Arni and colleagues focused on the setting parameters of EVLP. They proved that EVLP under subnormothermic temperature (28 °C) could improve the quality of rat donor lungs [19].

### 6.2. Immune Rejection and Immunosuppression Regimens

Innate and adaptive immune cells can induce rejection through indirect methods such as secreting cytokines and activating the complement system or directly by cytotoxicity. In addition, T lymphocytes can help B lymphocytes to produce antibodies and aggravate immune rejection [54]. The rat model is usually adopted in allotransplantation histology research by strain-mismatch such as allotransplantation from Brown Norway rat (BN) to Lewis (LEW) rat or from LEW to F344 rats [55,56,57]. While studies using the mouse model pay more attention to the immune cells and cytokines, e.g., Th17, γδ T cells, NK cells, and IL-17 [58,59,60]. Fan et al. established a reproducible model of chronic rejection by LTx from C57BL/10 mice to C57BL/6 (minor HLA mismatch model). They also found that neutralizing IL-17 can prevent chronic rejection, reduce acute rejection, and upregulate systemic IL-10 [61].

The classic immunosuppression regimen combination includes calcineurin inhibitors (e.g., tacrolimus or cyclosporin A), steroids (e.g., prednisone), and antimetabolites (e.g., mycophenolate) [62]. As mentioned in the introduction, the long-term usage of immunosuppression drugs could cause complications, including infection, nephrotoxicity, and solid organ tumors. Small animal models can also be used for research in this field for novel drugs research and improvement of adverse effects. Trametinib, a MEK pathway inhibitor, is usually used in cancer treatment such as malignant melanoma. In rats, LTx trametinib has been shown to attenuate graft rejection by suppressing T cell infiltration into the graft and its functional differentiation and B cell activation [63]. In addition, it has been demonstrated that the combined intravenous injection of adipose-derived mesenchymal stem cells and tacrolimus can improve histopathological evaluation, which could be a beneficial therapeutic approach after LTx [64].

## 7. Conclusions

Decades of research and development in the small animal model techniques increased the similarities to the human LTx procedure. This realistic simulation provides scientists with multiple opportunities to gain insights into the involvement of immune cells and the sequential steps in allograft failure and guide therapeutic discovery based on pathophysiological mechanisms. 

As mentioned above, rats and mice models are well balanced regarding cost–benefit ratio and feasible in their surgical complexity. Despite its incapability and limitation due to the biological characteristics and species barriers, the small animal model is likely to continue to play an essential role in LTx research and significantly contribute to the improvement of outcomes in human LTx.

## Figures and Tables

**Table 1:** Differences between large and small animal LTx models and between rats and mice.

	Large Animal Models	Small Animal Models	
	Porcine	Rats	Mice
Size	Large size: 40–50 kg	Medium size: 250–300 g	Smaller size: 20–30 g
Surgical complexity	Demanding surgical skills	Microsurgery training required	
Cost	High costs: purchase, housing	Lower costs	
Facility	Large facility, equipment, and housing	Easier to house, although surgical microscope required for procedure	
Anatomy	Closest related to humans	Larger evolutionary gap between rodents and humans	
Lifespan	Long lifespan	Short lifespan, fast metabolism rate, short gestation time	
Application	Surgical training, ex vivo lung perfusion, artificial lung	Complex applications: ex vivo lung perfusion, re-transplantation	Genetic modification: knock-out, knock-in, transgenic strains, etc.

**Table 2:** Comparison of anastomosis order and major outcomes.

Year	Author	Animal Strains	Sequence	Cuff/Suture	Transplantation Duration (min)	Survival
2013	Habertheuer et al. [33]	Male 250–300 g Fischer F344 rats to 320–350 g Wistar Kyoto rats	B-A-V	1 mm body 1 mm tail (PA&PV) 2 interrupted stabilization sutures (B)	90 ± 5	70–100% 2 w
2020	Tian et al. [25]	250–300 g Lewis or Brown Norway donor rats to Lewis rats	B-A-V	1.0 mm body, 1.5 mm tail	48.0 ± 2.8	97.2% 2 w
1982	Marck et al. [34]	250–300 g inbred Wistar Albino Glaxo and Brown Norway rats	V-A-B	Total interrupted sutures	87 (52–149)	40% 2 w
2004	Mizobuchi et al. [35]	male 250–300 g Fischer 344 rats to Wistar Kyoto rats	V-B-A	2.5 mm Body 1.5 mm Tail	84.8 ± 0.6	95.6% 2 w
2011	Rodríguez et al. [36]	male 300–400 g Sprague-Dawley consanguineous rats	A-B-V	1.5 mm Body 1.5 mm Tail	59.2 ± 4.2	80% 90-day
			PV/V—pulmonary vein; B—bronchus; PA/A—pulmonary artery			

## Data Availability

Not applicable.
